# Aurora A kinase is required for activation of the Fanconi anemia/BRCA pathway upon DNA damage

**DOI:** 10.1002/2211-5463.12087

**Published:** 2016-06-08

**Authors:** Min Jeong Chun, Soo Kyung Hwang, Hyoun Geun Kim, Sung‐Ho Goh, Sunshin Kim, Chang‐Hun Lee

**Affiliations:** ^1^ Cancer Cell and Molecular Biology BranchResearch InstituteNational Cancer CenterGoyangGyeonggiKorea; ^2^ Precision Medicine BranchResearch InstituteNational Cancer CenterGoyangGyeonggiKorea

**Keywords:** Aurora A kinase, DNA interstrand cross‐link lesions, Fanconi anemia/BRCA pathway

## Abstract

Previous studies have linked the DNA damage response to mitotic progression machinery. Mitotic kinases, such as Aurora A kinase and Polo‐like kinase, are involved in the phosphorylation of cell cycle regulators in response to DNA damage. Here, we investigated the potential involvement of Aurora A kinase in the activation of the Fanconi anemia (FA)/BRCA pathway, which participates in cellular response to DNA interstrand cross‐link lesions (ICL). Initially, we detected interactions between Aurora A kinase and FANCA protein, one of the components of the FA nuclear core complex. Silencing of Aurora A kinase led to inhibition of monoubiquitination of FANCD2 and formation of nuclear foci, the final consequences of FA/BRCA pathway activation upon ICL induction. An *in vitro* kinase assay revealed that Aurora A kinase phosphorylates S165 of FANCA. Moreover, this phosphorylation event was induced by the treatment with mitomycin C (MMC), an ICL‐inducing agent. In cells overexpressing S165A mutant FANCA, monoubiquitination of FANCD2 and nuclear foci formation was impaired and cellular sensitivity to MMC was enhanced. These results suggest that S165 phosphorylation by Aurora A kinase is required for proper activation of the FA/BRCA pathway in response to DNA damage.

**Abbreviations**
FAAP: FA‐associated proteins
FA: Fanconi anemia
FANC: FA complementation group proteins
HEK: human embryonic kidney
ICL: interstrand cross‐link lesions
MMC: mitomycin C

Fanconi anemia (FA) is an autosomal recessive human syndrome with diverse phenotypes of congenital abnormality, bone marrow failure, and cancer predisposition 1. To date, 19 genes encoding FA complementation group proteins (FANC) have been identified (Reviewed in 2). FANC proteins participate in the FA/BRCA signaling pathway 3 that is activated by DNA damage, predominantly DNA interstrand cross‐link lesions (ICL) 4. The FA nuclear core complex, comprising eight FANC proteins including FANCA, FANCL, and other FA‐associated proteins (FAAP), is activated upon DNA damage and induces monoubiquitination of FANCD2 protein via activity of FANCL E3 ligase 5, 6, 7. Monoubiquitinated FANCD2 forms nuclear foci in the region of DNA damage and participates in DNA damage repair in cooperation with BRCA1 4, 8.

FANC proteins have recently been shown to play an additional role during mitosis 9. Earlier reports demonstrate that these proteins are located in mitotic machinery, including centrosomes 10. Our group showed that FANCA protein localizes in centrosomes and is required for proper progression of mitosis 11, highlighting close crosstalk between the DNA damage response and mitotic machinery. Several publications have also linked the DNA damage response to mitosis regulation, including centrosome maintenance 12. For instance, the DNA damage response proteins, ATM 13, BRCA1 14, and p53 15, have been identified as centrosomal proteins, and their disruption results in numerical centrosomal aberrations and abnormal cell division, eventually leading to aneuploidy 16, 17. Conversely, mitotic protein kinases, such as Aurora A kinase and polo‐like kinase, participate in the DNA damage signaling pathway through phosphorylating cell cycle regulators such as Cdc25C 18, 19. Among these kinases, Aurora A kinase is a master regulator of mitotic progression required for proper modulation of centrosome maintenance and spindle assembly to ensure precise chromosomal segregation during mitosis 20.

In view of the possibility of close crosstalk between FA/BRCA pathway proteins and mitotic progression machinery, we analyzed the potential effects of Aurora A kinase on the FA/BRCA pathway. Our data clearly suggest that Aurora A kinase is a bona fide regulator of the FA/BRCA pathway, broadening our knowledge on the network of proteins involved in the close association between DNA damage response and regulation of mitotic progression.

## Materials and methods

### Cells

Human embryonic kidney (HEK) 293T and U2OS osteosarcoma cells were grown in Dulbecco's modified Eagle's medium (DMEM) supplemented with 10% FBS (Hyclone, Logan, UT, USA) at 37 °C.

### Coimmunoprecipitation

HEK 293T cells were lysed in lysis buffer (50 mm Tris–Cl (pH 7.4), 150 mm NaCl, 0.3% Igepal CA‐630, 0.2% Triton X‐100, 10 mm NaF, 1 mm sodium orthovanadate, and protease inhibitors). Cell lysates were incubated with 10 μL protein A‐agarose beads (Invitrogen, Carlsbad, CA, USA) for 1 h at 4 °C to remove nonspecific binding. Precleared lysates were incubated with anti‐FANCA antibody (A301‐980A; Bethyl Laboratories, Montgomery, TX, USA) for 18 h, followed by 10 μL protein A‐agarose beads for an additional 3 h at 4 °C. Beads were washed twice with lysis buffer and subjected to SDS/PAGE. The presence of Aurora A kinase was detected via immunoblotting with an anti‐Aurora A kinase antibody (Abcam, Cambridge, MA, USA).

### siRNA transfection

Transfection of synthetic siRNA into U2OS cells was performed using Lipofectamine 2000 reagent (Invitrogen), as described previously 11. Two siRNA targeting Aurora A kinase, specifically, Hs_AURKA_4 FlexiTube siRNA, and Hs_STK6_5 FlexiTube siRNA, were purchased from Qiagen, Valencia, CA, USA.

### Detection of FANCD2 monoubiquitination

To detect the mobility‐shifted monoubiquitinated FANCD2 band, lysate proteins were separated on 3–8% NuPAGE Tris‐Acetate gels (Invitrogen) and transferred to nitrocellulose membranes. FANCD2 was detected via immunoblotting with an anti‐FANCD2 antibody (Novus Biologicals, Littleton, CO, USA).

### Confocal microscopy

Confocal microscopy was performed as described previously 11. Briefly, U2OS cells were grown on coverslips (18 mm in diameter; Marienfeld Superior) in 12‐well plates, fixed in 3.7% formaldehyde in PBS for 20 min, permeabilized with 0.2% Triton X‐100 in PBS for 20 min, and blocked with 1% bovine serum albumin in PBST. U2OS cells on coverslips were sequentially incubated with anti‐FANCD2 (Novus Biologicals) overnight and Alexa 488‐conjugated donkey anti‐rabbit secondary antibody for 2 h. After extensive washing with PBST [Phosphate Buffered Saline supplemented with 0.2% Tween‐20], cells were counterstained with 4′,6‐diamidino‐2‐phenylindole (DAPI), mounted on glass slides, and observed under a Zeiss Axiovert LSM780 microscope (Carl Zeiss, Oberkochen, Germany). To count the number of foci, images were adjusted consistently by changing the gamma and white (brightness) parameters in the ZEN program (Carl Zeiss). After this adjustment, foci with pixel values of over 30 were counted.

### *In vitro* kinase assay

The *in vitro* kinase assay was performed using four previously described GST‐tagged FANCA fragments (#1 to #4) 11. Purified GST‐tagged FANCA fragments were incubated with 85 ng of Aurora A kinase protein (Cell Signaling Biotechnology, Danvers, MA, USA) in kinase buffer composed of 25 mm Tris–HCl (pH7.4), 10 mm MgCl_2_, 2 mm DTT, 200 μm cold ATP, and 5 μCi [γ‐P^32^] ATP (Perkin Elmer, Waltham, MA, USA) at 30 °C for 30 min. Samples were subjected to SDS/PAGE and transferred to nitrocellulose membranes (GE Healthcare, Milwaukee, WI, USA), followed by autoradiography to detect phosphorylated protein.

### Site‐specific mutagenesis

Site‐specific mutagenesis was performed using the QuikChange kit (Stratagene, La Jolla, CA, USA) with oligonucleotides incorporating the S165A or T256A mutation. The template plasmids used were pGEX‐FANCA#1 and pcDNA3‐HA‐FANCA 11. Primer sequences were as follows: 5′‐GTATGTTCTCCCGTCTTtCCTTCTGTCAAGAATTATGG‐3′ (S165A_F), 5′‐CCATAATTCTTGACAGAAGGaAAGACGGGAGAACATAC‐3′ (S165A_R), 5′‐GATCTGAGAAGAaCTGTGGAGCCTGAAAAAATGCC‐3′ (T256A_F), and 5′‐GGCATTTTTTCAGGCTCCACAGtTCTTCTCAGATC‐3′ (T256A_R).

### Generation of phospho‐specific antibody

Rabbits were immunized with phosphopeptide containing the Ac‐MFSRL‐pS‐FC sequence (Peptron, Daejeon, Korea). After the second boost, antiserum was collected and phospho‐specific antibodies were purified using affinity chromatography with phosphopeptide‐conjugated resin. Bound antibody was eluted in 100 mm glycine (pH 2.5), subsequently neutralized by the addition of 1 m Tris.

### Immunoprecipitation–western blotting

HEK293T cells were transiently transfected with pcDNA3‐HA‐FANCA‐WT or S165A using Effectene transfection reagent (Qiagen). Cell lysates were immunoprecipitated with 0.3 μg phospho‐specific S165 antibody (P‐S165), and immunoprecipitates were subjected to electrophoresis on 3–8% NuPAGE Tris‐acetate gels (Invitrogen). FANCA was detected via immunoblotting with HRP‐conjugated anti‐HA antibody (Santa Cruz Biotechnology Inc., Santa Cruz, CA, USA) or anti‐FANCA antibody (Bethyl Laboratories). The presence of equal amounts of FANCA for immunoprecipitation was confirmed by immunoblotting of input lysate.

### Establishment of FANCA‐expressing stable cell lines

U2OS cells stably expressing S165A mutant FANCA were generated via transfecting with pcDNA3‐HA‐FANCA‐WT or ‐S165A. At 2 days post‐transfection, G418 sulfate (Invitrogen) was added at a concentration of 1 mg·mL^−1^. Selection of stable transfectants was performed until distinct colonies emerged. After propagation of resistant colonies, FANCA‐expressing clones were confirmed by immunoblotting with anti‐HA antibody.

## Results

### Aurora A kinase interacts with FANCA

**Figure 1:**
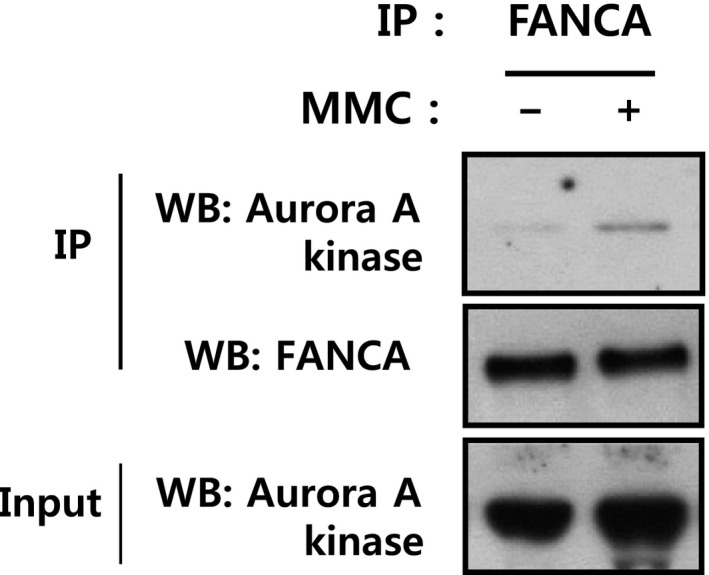
Interaction of FANCA with Aurora A kinase. HEK 293T cells were treated with 200 ng·mL^−1^ MMC for 16 h. Cell lysates were immunoprecipitated with anti‐FANCA antibody and Protein A‐conjugated agarose. The presence of Aurora A kinase (top panel) and immunoprecipitated FANCA (middle panel) in immunoprecipitates was assessed by immunoblotting with the respective antibodies. The presence of equal amounts of Aurora A kinase in inputs was confirmed (bottom panel).

To determine whether Aurora A kinase participates in regulation of the FA/BRCA pathway, a coimmunoprecipitation assay was initially performed to detect FANCA interactions with Aurora A kinase. FANCA was precipitated with a specific antibody, and the presence of Aurora A kinase in immunoprecipitates assessed via western blot. Our results clearly revealed interactions between FANCA and Aurora A kinase after treatment with mitomycin C (MMC), a DNA cross‐linking agent (Fig. 1). The observed binding between FANCA and Aurora A kinase supports the potential involvement of Aurora A kinase in activation of the FA/BRCA pathway in response to DNA damage.

### Knockdown of Aurora A kinase impairs activation of the FA/BRCA pathway upon DNA damage

**Figure 2:**
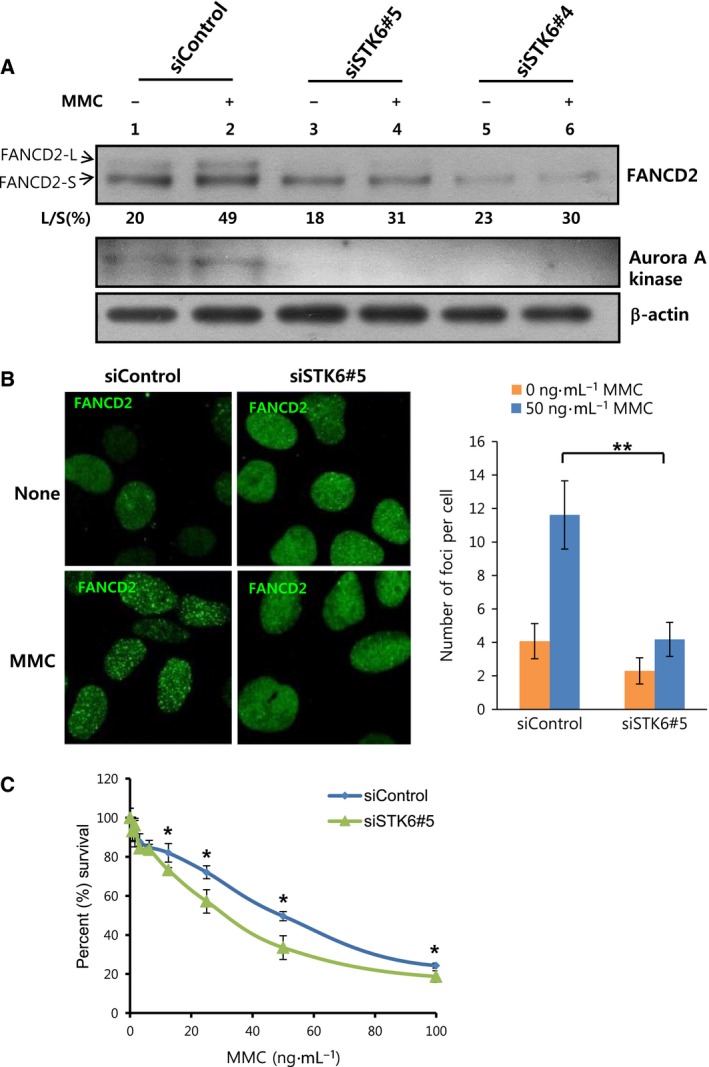
Aurora A kinase is required for the activation of the FA/BRCA pathway in response to DNA interstrand cross‐linking lesions. (A) Knockdown of Aurora A kinase abolishes MMC‐induced FANCD2 monoubiquitination. After transfection with siRNA for Aurora A kinase (siSTK6#5 and siSTK6#4) or control siRNA (siControl), U2OS cells were treated with 25 ng·mL^−1^ MMC for 8 h. Mobility‐shifted monoubiquitinated FANCD2 (FANCD2‐L) and unmodified FANCD2 (FANCD2‐S) were visualized by immunoblotting with anti‐FANCD2 (top panel). The ratios (L/S) of band intensities of FANCD2‐L and FANCD2‐S are shown. Levels of Aurora A kinase (middle panel) and β‐actin (bottom panel) were assessed to confirm knockdown. (B) Knockdown of Aurora A kinase suppresses the formation of FANCD2 nuclear foci upon MMC treatment. After siRNA(siControl or siSTK6#5) transfection, cells were treated with 50 ng·mL^−1^ MMC for 8 h and stained with anti‐FANCD2 (green) for confocal microscopy. Representative images are shown in the left panels and the number of foci per cell was counted for over 20 cells and plotted in the right panel. Values represent means ± SEMs. (Student's *t*‐test, **P < 0.01). (C) Cellular sensitivity to MMC is enhanced after Aurora A kinase knockdown. After siRNA transfection, cells were cultured in 96‐well plates and treated with serial two‐fold dilutions of MMC (from 100 ng·mL^−1^) in triplicate for 4 days. Cell viability was measured with the MTT assay and percentage survival was calculated, compared with untreated cells. A representative graph from three independent experiments is shown. Values represent means ± SDs. (Student's *t*‐test, *P < 0.05).

To confirm the participation of Aurora A kinase in activation of the FA/BRCA pathway, we suppressed Aurora A kinase expression via siRNA transfection in U2OS cells and subsequently induced DNA damage by treating cells with MMC. MMC‐induced FANCD2 monoubiquitination, the final consequence of FA/BRCA pathway activation that is readily detected as a mobility‐shifted band on western blot, was reduced following knockdown of Aurora A kinase (Fig. 2A). The ratios (L/S) of the band intensities of monoubiquitinated FANCD2 (FANCD2‐L) and unmodified FANCD2 (FANCD2‐S) decreased from 49% (lane 2) to 31% and 30% (lanes 4 and 6, respectively) after Aurora A kinase knockdown. Formation of FANCD2 nuclear foci in response to MMC treatment also decreased upon Aurora A kinase knockdown (Fig. 2B). In this experiment, the number of foci in siSTK6#5‐transfected cells was only 36% of that in siControl‐transfected cells. Furthermore, cellular sensitivity to MMC was enhanced, as measured using the 3‐(4,5‐dimethylthiazol‐2‐yl)‐2,5‐diphenyl‐tetrazolium bromide (MTT) assay (Fig. 2C). In this experiment, survival of siSTK6#5‐transfected cells was decreased by statistically significant extent, compared with that of siControl‐transfected cells. Our results indicate that Aurora A kinase is required for proper activation of the FA/BRCA pathway upon DNA damage.

### Phosphorylation of FANCA by Aurora A kinase

**Figure 3:**
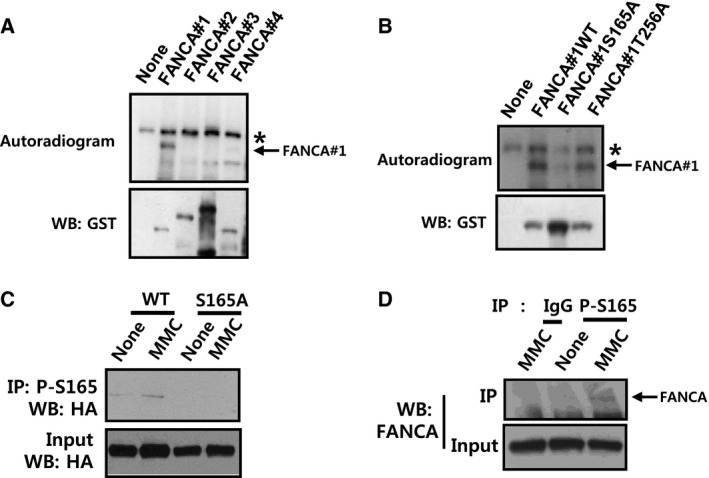
Phosphorylation of FANCA by Aurora A kinase. (A) Phosphorylation of the N terminus of FANCA by Aurora A kinase was detected using the *in vitro* kinase assay. GST‐tagged FANCA fragments (GST‐FANCA#1, #2, #3 and #4) were incubated at 30 °C for 30 min with recombinant Aurora A kinase in the presence of [γ‐^32^P] ATP. After separation via SDS/PAGE and transfer onto nitrocellulose membrane, phosphorylated proteins were visualized using autoradiography (top panel). The position of phosphorylated GST‐FANCA#1 is indicated by an arrow. Autophosphorylated AMPK is marked with an asterisk. Levels of substrates were verified by immunoblotting with anti‐GST antibody (bottom panel). (B) Determination of the phosphorylation site via site‐specific mutagenesis and *in vitro* kinase assay. GST‐FANCA#1 constructs with potential phosphorylation sites mutated to alanine (S165A and T256A) were generated and used for an *in vitro* kinase assay, as described for A. (C) Detection of FANCA phosphorylated at S165 with immunoprecipitation (IP)–western blotting using a phospho‐specific antibody. HEK 293T cells were transfected with pcDNA3‐HA‐FANCA‐WT or pcDNA3‐HA‐FANCA‐S165A, followed by treatment with 100 ng·mL^−1^ of MMC. Cell lysates were immunoprecipitated with phospho‐specific antibody recognizing S165‐phosphorylated FANCA (P‐S165), and the presence of HA‐FANCA was detected with an anti‐HA antibody (top panel). The presence of equal amounts of HA‐FANCA in starting samples was verified by immunoblotting of inputs (bottom panel). (D) Detection of endogenous FANCA phosphorylated at S165. HEK 293T cells were treated with 100 ng·mL^−1^ of MMC, and IP–western blotting was performed using P‐S165 antibody. The presence of endogenous FANCA was detected with anti‐FANCA antibody (top panel). The presence of equal amounts of FANCA in inputs was confirmed at the bottom panel.

Next, we attempted to clarify the mechanism underlying the requirement for Aurora A kinase in FA/BRCA pathway activation. To achieve this goal, we examined the possibility that FANCA protein is directly phosphorylated by Aurora A kinase. Results from the *in vitro* kinase assay using GST‐tagged FANCA fragments (FANCA#1, aa#1‐375; FANCA#2, aa#331‐736; FANCA#3, aa#691‐1153; FANCA#4, aa#1083‐1455) showed phosphorylation of the N‐terminal portion of FANCA protein (FANCA#1) by Aurora A kinase (Fig. 3A). On the basis of the consensus phosphorylation sequence of the kinase 21, we identified two potential phosphorylation sites within this region, S165 and T256. In GST‐FANCA#1 mutants with alanine substitutions at the prospective phosphorylation sites (S165A and T256A), phosphorylation was abolished for the S165A but not T256A mutant, suggesting that S165 is the specific target of Aurora A kinase (Fig. 3B).

Next, we raised a phospho‐specific antibody for S165 of FANCA (P‐S165) to check whether S165 phosphorylation would occur in cells. In these experiments, we used HEK 293T cells, which have a high transfection efficiency and a high level of FANCA expression. Through immunoprecipitation (IP)–western blot experiments after the overexpression of HA‐tagged FANCA, the presence of the S165 phosphorylation band was confirmed in wild‐type (WT) HA‐FANCA‐transfected cells (Fig. 3C). Band intensity was enhanced in MMC‐treated samples, indicating that the phosphorylation event is induced upon DNA damage. No bands were detected in samples prepared from cells transfected with the S165A mutant of HA‐FANCA, confirming the specificity of the phospho‐specific antibody. These data clearly suggest that S165 of FANCA is phosphorylated by Aurora A kinase within cells. Similar induction of S165 phosphorylation upon MMC treatment was detected in endogenous FANCA (Fig. 3D).

### Involvement of S165 phosphorylation in MMC‐induced FANCD2 monoubiquitination

**Figure 4:**
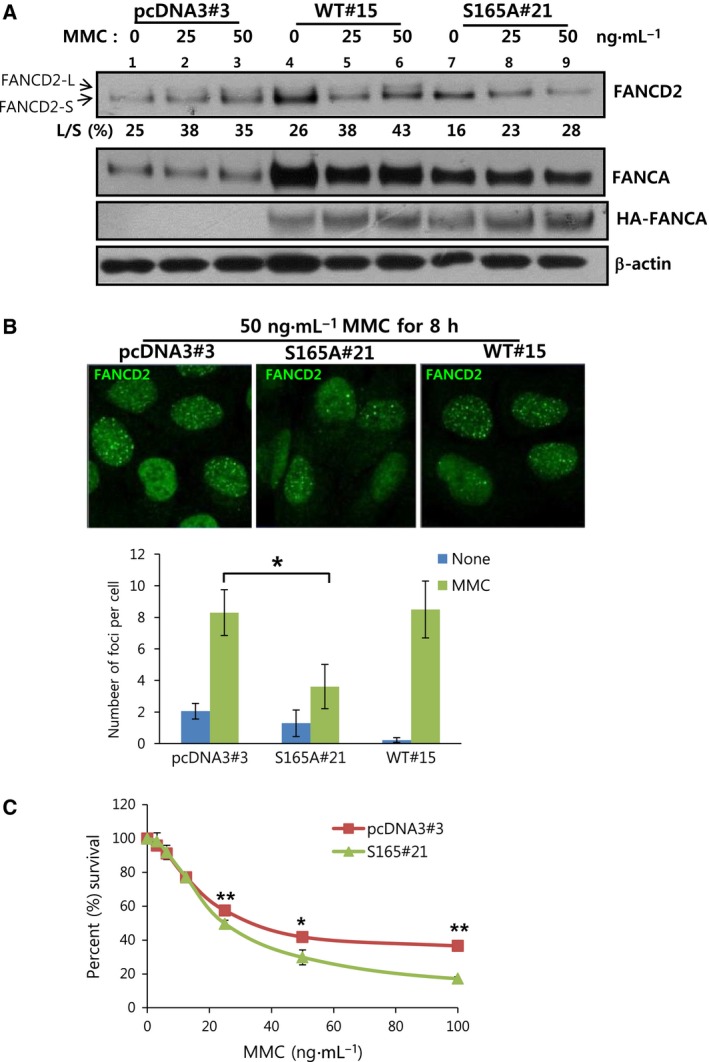
Functional significance of S165 phosphorylation in FA/BRCA pathway activation. (A) FANCD2 monoubiquitination upon MMC treatment is abolished in the S165A FANCA‐overexpressing U2OS cell line. U2OS cells stably overexpressing S165A (S165A#21) or WT HA‐FANCA (WT#15) were treated with 25 or 50 ng·mL^−1^ MMC for 8 h. FANCD2 monoubiquitination (FANCD2‐L) was detected with anti‐FANCD2 immunoblotting (top panel). Overexpression of HA‐FANCA was verified by immunoblotting with anti‐FANCA and anti‐HA (middle panels). Empty vector‐transfected cells (pcDNA3#3) were also included in the experiment. (B) Formation of FANCD2 nuclear foci upon MMC treatment is decreased in S165A‐expressing stable cells. Cells were treated with 50 ng·mL^−1^ MMC for 8 h and stained with anti‐FANCD2 (green) for confocal microscopy. Representative images are shown in upper panels and the number of foci per cell was counted and plotted in lower panel. Values represent the means ± SEM. (Student's *t*‐test, *P < 0.05). (C) MMC sensitivity of S165A‐overexpressing cells is enhanced. The percentage survival of cells was measured as described for Fig. 2C. A representative graph from three independent experiments is shown. Values represent means ± SDs. (Student's *t*‐test, *P < 0.05; **P < 0.01).

We further investigated the functional significance of FANCA S165 phosphorylation in response to MMC treatment by establishing U2OS cell lines permanently expressing S165A mutant FANCA (S165A#21), empty vector control (pcDNA3#3) or wild‐type FANCA (WT#15). The expression of HA‐FANCA in these cells were confirmed by immunoblotting with anti‐FANCA and anti‐HA antibodies (Fig. 4A). MMC‐induced FANCD2 monoubiquitination (FANCD2‐L), clearly observed in pcDNA3#3 (lane 3) and WT#15 (lane 6) cell lines, was impaired in S165A#21 cells (lane 9). The formation of FANCD2 nuclear foci upon MMC treatment was also abolished in S165A#21 cells (Fig. 4B). Cellular sensitivity to MMC was enhanced in these cells, compared with WT FANCA‐expressing cells (Fig. 4C). On the basis of the collective findings, we propose that the S165 residue of FANCA is important for activation of the FA/BRCA pathway upon MMC treatment, possibly via Aurora A kinase‐induced phosphorylation.

## Discussion

In this study, we showed that Aurora A kinase, a well‐known mitotic kinase, is required for FA/BRCA pathway activation in response to DNA damage through direct phosphorylation of FANCA at S165. Although the function of Aurora A kinase in the regulation of mitotic progression has been well established, the requirement of Aurora A kinase for FA/BRCA pathway activation is not likely to be restricted to the mitotic phase of the cell cycle. First, MMC treatment induces cell cycle arrest mainly at S and G2 phase 22. Second, studies have shown the involvement of Aurora A kinase in DNA damage response modulating cellular radio‐ and chemoresistance through the ATM/chk2‐mediated DNA repair network 23, 24, which is activated mainly during interphase.

Several protein kinases are implicated in FA/BRCA pathway activation. For example, ATR has been extensively characterized as a kinase phosphorylating several FANC proteins, including FANCA, and is required for proper FA/BRCA pathway activation 25, 26, 27. ATM is also implicated in the S phase‐specific phosphorylation of FANCD2 upon exposure to ionizing radiation 28. Another DNA damage response and checkpoint kinase, Chk1, is proposed to be required for FA/BRCA pathway activation through FANCE phosphorylation 29. Confining our discussion to FANCA phosphorylation events, FANCA has been reported to be phosphorylated on S1449 by ATR upon DNA damage 26. This phosphorylation is induced by MMC treatment but not during S phase 26, which is different from other post‐translational modifications of FANC proteins, including FANCD2 monoubiquitination 8. Further studies are needed to determine whether S165 phosphorylation by Aurora A kinase also occurs during S phase. Moreover, future studies should seek to elucidate the relationships between phosphorylation events by ATR and Aurora A kinase. For this, the effects of ATR knockdown and/or S1449A mutation on S165 phosphorylation might give valuable information on the mechanism underlying FANCA phosphorylation upon DNA damage. In this regard, it is interesting that Aurora A kinase suppressed the expression of ATR in breast and ovarian cancer cells 23.

The discovery that a well‐known mitotic kinase is involved in the FA/BRCA DNA damage‐signaling pathway in this study further highlights the close relationship between DNA damage response and mitotic progression. The underlying reason for crosstalk between DNA damage response and mitotic progression may be the requirement for coordination of all phases of cell cycle progression. In this regard, the centrosome plays a pivotal role as a signaling center of cell cycle coordination, since the centrosome duplication cycle itself must be well coordinated with cell cycle progression. During this coordination process, Aurora A kinase may play dual roles in response to ICL DNA damage: one to exert effects on centrosome duplication and mitotic progression and the other to directly regulate the cellular response and cell cycle checkpoint activation upon DNA damage. This type of dual role is a characteristic of FANC proteins. FANC proteins, including FANCA, are suggested to function in ensuring proper progression through the mitotic phase of the cell cycle, mainly localizing to centrosomes 9, 10, 11. In support of the close relationship between FANC proteins and mitotic kinases, FANCJ is proposed to regulate ICL‐induced centrosome amplification through polo‐like kinase (PLK) activation 30.

Aurora A kinase is frequently overexpressed in human cancers 31, 32, 33, and has been targeted in several clinical trials for anticancer therapy, either as monotherapy or in combination with other conventional chemotherapeutic drugs 34. Data from the current study provide a platform highlighting the potential utility of Aurora A kinase as a target for enhancing the anticancer efficacy of ICL‐inducing drugs such as cisplatin.

## Conclusions

This study has provided evidence supporting the requirement of Aurora A kinase, a key regulator during mitotic progression, in the activation of the FA/BRCA pathway upon induction of DNA interstrand cross‐link lesions. Aurora A kinase has been confirmed to phosphorylate the S165 residue of FANCA of the FA/BRCA pathway. We have also presented data suggesting that the S165 residue is important for proper activation of the FA/BRCA pathway.

## Author contributions

CHL conceived and designed the experiments; MJC, SKH, HGK, and CHL performed the experiments; SHG, SK, and CHL analyzed the data; SHG, SK and, CHL wrote the manuscript.
